# Temporal trends in the epidemiology of inflammatory bowel diseases in the public healthcare system in Brazil: A large population-based study

**DOI:** 10.1016/j.lana.2022.100298

**Published:** 2022-06-09

**Authors:** Abel B. Quaresma, Aderson O.M.C. Damiao, Claudio S.R. Coy, Daniela O. Magro, Adriano A.F. Hino, Douglas A. Valverde, Remo Panaccione, Stephanie B. Coward, Siew C. Ng, Gilaad G. Kaplan, Paulo G. Kotze

**Affiliations:** a Universidade do Oeste de Santa Catarina, UNOESC, Colorectal Surgery, Joaçaba, Brazil; b University of Sao Paulo, Gastroenterology, São Paulo, Brazil; c University of Campinas UNICAMP, Colorectal Surgery Unit, Campinas, Brazil; d Catholic University of Paraná, Health Sciences Postgraduate Program, Curitiba, Brazil; e Techtrials Healthcare, Data Science, Vinhedo, Brazil; f University of Calgary, Department of Medicine, Division of Gastroenterology and Hepatology, Calgary, Canada; g Department of Medicine and Therapeutics, Institute of Digestive Disease, State Key Laboratory of Digestive Diseases, LKS Institute of Health Sciences, The Chinese University of Hong Kong, Hong Kong, China; h Catholic University of Paraná, IBD outpatient Clinics, Colorectal Surgery Unit, Curitiba, Brazil

**Keywords:** Incidence, Prevalence, Inflammatory bowel disease, Ulcerative colitis, Crohn's disease, Epidemiology, Brazil, AC, Acre, AL, Alagoas, AP, Amapá, AM, Amazonas, AAPC, Average Annual Percent Change, BA, Bahia, CE, Ceará, CAAE, Certificate of Presentation for Ethical Appreciation, ICD-10, Classification of Diseases and Related Health Problems, Tenth Revision, CI, confidence intervals, CD, Crohn's disease, DATASUS, Department of Health Informatics/Ministry of Health, DF, Distrito federal, ES, Espírito Santo, GO, Goiás, IBD, Inflammatory Bowel Diseases, IBDU, Inflammatory Bowel Diseases undetermined, MA, Maranhão, MT, Mato Grosso, MS, Mato Grosso do Sul, MG, Minas Gerais, IBGE, National Institute of Geographics and Statistics (*Instituto Brasileiro de Geografia e Estatística)*, SUS, national public health system (*Sistema Único de Saúde)*, PA, Pará, PB, Paraíba, PR, Paraná, PE, Pernambuco, PI, Piauí, RN, Rio Grande do Norte, RS, Rio Grande do Sul, RO, Rondônia, RR, Roraima, SC, Santa Catarina, SP, São Paulo, SE, Sergipe, TO, Tocantins, UC, ulcerative colitis, UNOESC, University of the West of Santa Catarina

## Abstract

**Background:**

Population-based data on epidemiology of Inflammatory Bowel Diseases (IBD) in Brazil are scarce. This study aims to define temporal trends of incidence and prevalence rates of Crohn's disease (CD) and ulcerative colitis (UC) in Brazil.

**Methods:**

All IBD patients from the public healthcare national system were included from January 2012 to December 2020. Average Annual Percent Change (AAPC) and 95% confidence intervals (CI) were calculated using log-linear regression for incidence and binomial regression for prevalence. Moran's I autocorrelation index was used to analyse clustering of cities by level of prevalence.

**Findings:**

A total of 212,026 IBD patients were included. Incidence of IBD rose from 9.4 in 2012 to 9.6 per 100,000 in 2020 (AAPC=0.8%; 95% CI -0.37, 1.99); for UC, incidence increased from 5.7 to 6.9 per 100,000 (AAPC=3.0%; 95% CI 1.51, 4.58) and for CD incidence decreased from 3.7 to 2.7 per 100,000 (AAPC=-3.2%; 95% CI -4.45, -2.02). Prevalence of IBD increased from 30.0 in 2012 to 100.1 per 100,000 in 2020 (AAPC=14.8%; CI 14.78-14.95); for UC, from 15.7 to 56.5 per 100,000 (AAPC=16.0%; CI 15.94, 16.17); for CD from 12.6 to 33.7 per 100,000 (AAPC=12.1% CI 11.95, 12.02). A south-north gradient was observed in 2020 prevalence rates of IBD [I=0.40 (p<0.0001)], CD [I=0.22 (p<0.0001)] and UC [I=0.42 (p<0.0001)].

**Interpretation:**

Incidence of CD is decreasing whereas of UC is increasing, leading to stabilization in the incidence of IBD from 2012 to 2020 in Brazil. Prevalence of IBD has been climbing with 0.1% of Brazilians living with IBD in 2020.

**Funding:**

None.

**Research in context**

**Evidence before the study**

There is lack of high quality, national, population-based epidemiological data from newly industrialized countries globally, and temporal trends of incidence and prevalence rates are lacking in these specific areas of the world.

**Added value of the study**

Incidence rates of IBD have remained stable in Brazil from 2012-2020. Incidence of CD is decreasing whereas of UC is increasing. There was a significant increase in cumulative prevalence rates of CD and UC over the nine years of the study period. Prevalence rates in 2020 were higher in the south and southeastern regions as compared to north and northeastern, determining a south-north gradient of IBD cases.

**Implications of all the available evidence**

The significant rise in cumulative prevalence of IBD in Brazil can support planning for future strategies for public healthcare providers in our country towards better IBD care.

Alt-text: Unlabelled box

## Introduction

Recent reviews have described global epidemiological trends of incidence and prevalence of IBD over the last decades.^1^^,^^2^ The evolution of both Crohn's disease (CD) and ulcerative colitis (UC) can be stratified in four epidemiological stages: emergence of new cases, acceleration in incidence, compounding prevalence and prevalence equilibrium.^3^

Currently, newly industrialized countries in Latin America are considered to be in the phase of acceleration in incidence with rapidly rising incidence, but lower prevalence of IBD. In contrast, countries of the Western world (e.g., North America and Northern Europe) are in the compounding prevalence phase with stabilization in incidence, but escalation in the prevalence of IBD. In recent years, changes have been observed in the classic geographic distribution of IBD, with increasing rates of incidence and prevalence in traditionally low-incidence regions, such as Asia, South America, and Eastern Europe.^4^, ^5^, ^6^, ^7^ Despite the lack of population-based national data from Latin America, incidence and prevalence of IBD are increasing, affecting young individuals in more urbanized and industrialized societies, indicating IBD's emergence as a global disease.^1^^,^^2^

To date, there is lack of high quality, national, population-based epidemiological data from newly industrialized countries in Latin America, and temporal trends of incidence and prevalence rates are lacking in these specific areas of the world. Brazil is the largest newly industrialized country from Latin America, with the largest population and a historical heterogeneous background of mixed colonization (Europeans, Middle-Easterners and Asians).^8^ In recent systematic reviews, it was considered a country with low to intermediate incidence and prevalence rates of IBD.^2^ An increase in the incidence and prevalence rates of CD and UC has been observed in the last decades.^9^, ^10^, ^11^, ^12^, ^13^, ^14^ However, to date, there remains a lack of national level data or comparison across the diverse geographic heterogeneity of Brazil.

The aim of this population-based study is to describe temporal trends of national incidence and prevalence rates of IBD in the Brazilian unique public health system between 2012 and 2020, demonstrating possible internal differences between different regions of the country.

## Methods

### Data source

Publicly available data were extracted from the Department of Health Informatics/ Ministry of Health (DATASUS) (http://www2.datasus.gov.br/DATASUS) using the TT Disease Explorer platform, developed by Techtrials^TM^ (Techtrials Healthcare Data Science, Brazil) which continuously collects epidemiological data and other information such as diagnostic and therapeutic interventions.^15^ The database is automatically updated using Microsoft Azure, SQL, M and DAX coding, and Power BI visualization tools (Microsoft Corporation, Redmond, Washington, USA). This platform automatically collects through artificial intelligence (ETLs and Webcrawlers) structured health data which is publicly available. The collected data were derived from the national public health system (*Sistema Único de Saúde* - SUS) and are encrypted and translated by a specific tool which does not allow duplication of patients, who have a unique identification number. If the same patient performs procedures in different places of the country, the platform identifies this and avoids duplicates.

DATASUS is an open access population-based health and disease registry that contains information from the national unified health system (SUS) on medical procedures, hospital admissions, discharges, mortality and demographic variables.^15^ It covers roughly the whole population of the country, as every citizen has the right to use the national public health system. All data are anonymous and do not allow identifying individual subjects, however, de-identified individuals can be tracked throughout the database across time through their unique national health identification number. Currently, the Brazilian SUS is one of the largest public health systems in the world and the Brazilian average number of beds per 1,000 inhabitants is 2.3. Considering only SUS beds, this ratio drops to 2 and in the private healthcare system, it rises to 3.5.^16^ A complete analysis of the distribution of 493,010 beds in all Brazilian hospitals (National Register of Health Establishments - CNES, 2019) indicates that 66.6% exclusively serve the public system (SUS), for 77.8% of the Brazilian population. The remaining 22.2% of the population (with some type of private health insurance) have access to 33.4% of the total available beds.^17^ The subset of the Brazilian population accessing private healthcare outside of SUS may overlap with the national healthcare, particularly when prescribed costly medications such as biologics, and thus SUS likely captures the majority of individuals with IBD living in Brazil.

Records of hospital admissions, outpatient procedures, consultations and IBD-related medication dispensing obtained from the DATASUS registry were searched according to the International Statistical Classification of Diseases and Related Health Problems, Tenth Revision (ICD-10). The International Classification of Diseases, codes K50 and K51 and their respective subtypes (K50.0, K50.1, K50.8, K50.9, K51.0, K51.1, K51.5, K51.8 and K51.9) were used to identify patients with CD or UC, respectively.

### Study design, population, and variables

An observational and population-based study was carried out with DATASUS records from those with IBD from January 1st, 2012 to December 31st, 2020. This platform has data as of 2008. We took the data from 2012, forcing a 4-year retroactive elimination period (washout) to ensure that new diagnosis of IBD occurrence were not mixed with prevalent cases for the inception cohort. The study period was also selected based on the most recent and standardized data available, beginning in the first full fiscal year of electronic registration of data (2012). A total of 9 years of full data was extracted. All patients who started any IBD-related treatment, who underwent any consultation, diagnostic or surgical procedure with any of the IBD ICD codes were included, provided they appear at least twice in the system (two codes in the analysis period). The two code minimum was used to reduce misclassification error, as employed in other electronic medical record databases.^18^ Data extraction was performed by the platform and checked by two independent reviewers. Individuals who only had codes for CD or UC were classified accordingly. Individuals with codes for both UC and CD were classified as undetermined (IBDU).

The date of first contact of an IBD code in the health system was defined as the index date for IBD. To define the incidence cohort, all records in the DATASUS dating back to 2008 were searched to confirm no prior code of IBD. Clinical and demographic data, such as age and sex, were collected at the time of the index date. To determine the point prevalence (existing cases/ 100,000 inhabitants/ period), patients who used the system with IBD codes between January 1st, 2012 and December 31st, 2020 were included, and data for each year was calculated. To determine the incidence (new cases/ 100,000 inhabitants/ period), patients with first code of IBD from January 1^st^ to December 31^st^ of each fiscal year were analysed separately.

Our denominator was the Brazilian total population estimated by the National Institute of Geographics and Statistics (*Instituto Brasileiro de Geografia e Estatística* – IBGE).^19^

Variables captured in the electronic forms include demographic data, medications used, and procedures performed during hospitalization or in outpatient basis. Age groups were stratified every 5 years, from zero to over 100 years. The variables analysed were age, sex, city of origin, date of entry and exit in the registration system and disease type (UC or CD). To define the geographic distribution, we used two criteria: division by macro-regions (North, Northeast, Southeast, South and Midwest) and division by 5,565 municipalities, according to IBGE. To analyse the geographic distribution, the prevalence rates of IBD per 100,000 inhabitants were calculated in each municipality.

### Statistical analysis

Age and sex at index date were demonstrated in a descriptive analysis. The average annual percentage change (AAPC) and the 95% confidence intervals (CI) were calculated using Poisson regression (or negative binomial) for incidence rates and log binomial regression for prevalence rates, with STATA v.16 software (Statacorp LLC, College Station, TX, USA). Exploratory procedures with a quantitative approach were applied to the data using IBM SPSS software for Windows (v.20, SPSS Inc., Chicago, IL, USA).

### Mapping and group analysis

The development of incidence and prevalence maps of IBD, CD and UC in the states of the federation was based on quintiles. To analyse the IBD, CD and UC prevalence among the municipalities on Brazilian territory, we used the Moran's I spatial autocorrelation analysis. The Global Moran's I index varies from -1,0 to +1,0 and indicate whether the spatial pattern is clustered (differ from 0) or randomly distributed (close to 0). The 5,565 municipalities were considered the unit in the Moran's I analysis. Once in the Geographic Information System they are represented as polygons, the variable of interest is continuous (prevalence of IBD, CD and UC) and we expect as the municipalities share boundaries the spatial interaction between them increases, the spatial relationship was set as a first order Contiguity Edges Corners.^20^

If a clustered pattern was identified, the Hot Spot analysis was used to identify clusters of spots with highest prevalence (“hot areas”) and lowest prevalence (“cold areas”) of IBD, CD and UC considering a confidence interval of 95%. The confidence interval is derived from the z-score and p-value computed considering each municipality within the context of neighbouring municipalities and determining if the local pattern (a target municipality and the neighbour municipalities) is statistically different from the global pattern.^20^ All spatial statistics analyses were conducted using ArcGIS v.10.3, considering a level of statistical significance of p<0.05 and False Discovery Rate correction, a more conservative approach, to account for multiple testing and spatial dependency.^21^

### Ethical considerations

This study was approved by the Research Ethics Committee of the University of the West of Santa Catarina (UNOESC), in accordance with the Certificate of Presentation for Ethical Appreciation (CAAE) 97905918.7.0000.5367. During all stages of this research, all guidelines of Resolution 466/2012 of the National Health Council were strictly followed and complied.

### Role of the funding source

None.

## Results

**Figure 1:**
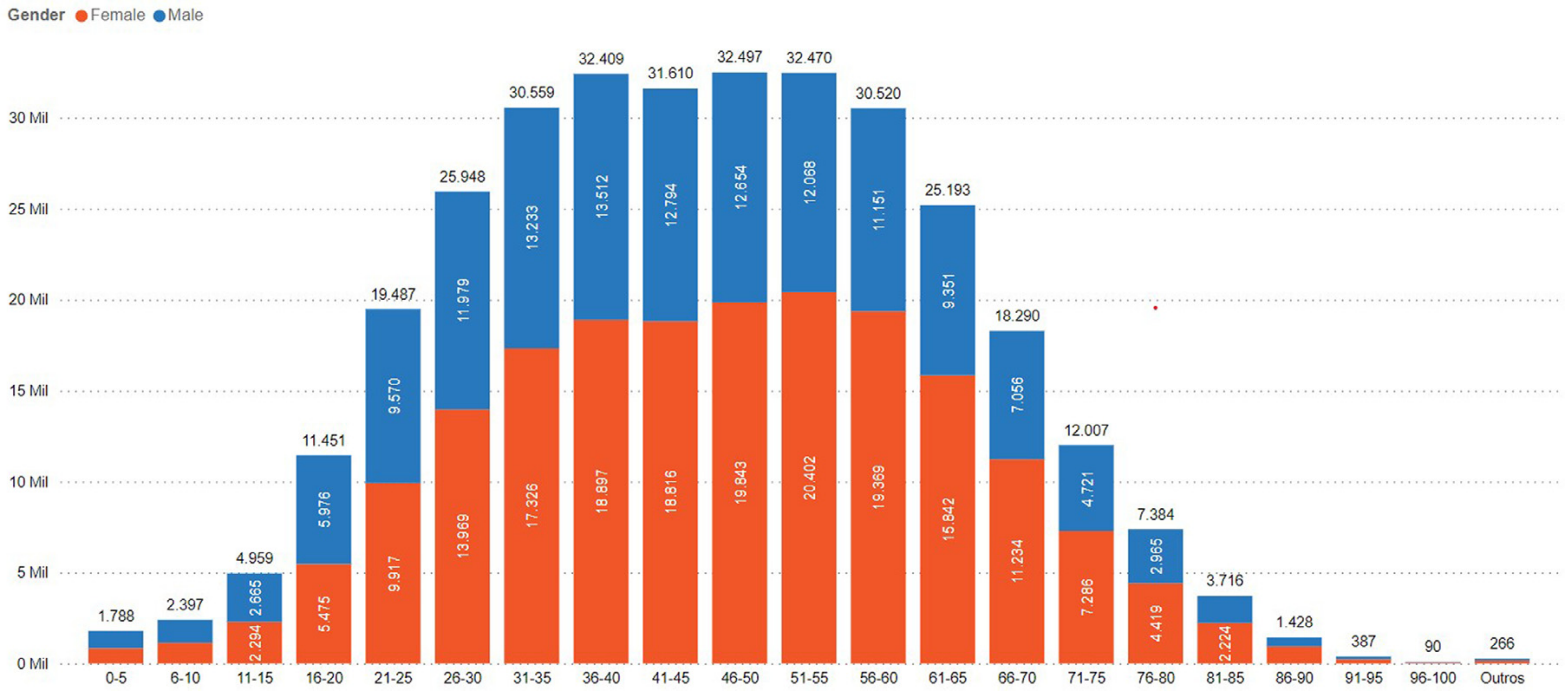
IBD patients in Brazil by age group at index date and sex.

A total of 212,026 unique IBD patients were identified, (UC: n=119,700; CD: n=71,321; IBDU: n=21,005). The UC:CD ratio was 1.7:1. There was a predominance of females (n=123,992, 58.5%) as compared to male patients (n=86,902, 41%), and 0,5% had undetermined sex (n=1,132 patients). The age at index date was most common between 36 and 55 years (Figure 1).

**Figure 2:**
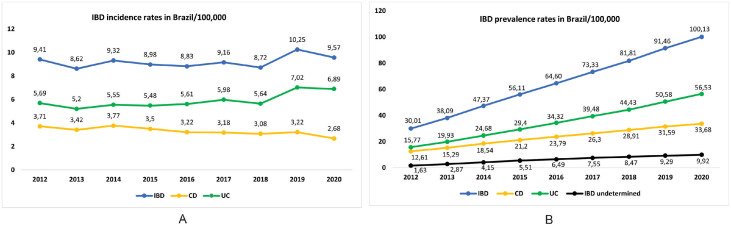
Temporal trends of IBD, CD and UC in Brazil in the public health system from 2012-2020. A: incidence; B: prevalence with IBDU.

The incidence rates of IBD remained stable from 2012 to 2020 [9.4/100,000 inhabitants in 2012 and 9.6/100,000 in 2020 (AAPC=0.8%; 95% CI -0.37 - 1.99; p=0.1801)]. In UC, incidence rates increased significantly from 5.7/100,000 to 6.9/100,000 (AAPC=3.0%; 95% CI 1.51 - 4.58; p<0.0001). In CD, incidence rates dropped significantly from 3.7/100,000 to 2.7/100,000 (AAPC= -3.2%; 95% CI -4.45 - -2.02; p<0.0002) in the same period. These data are shown in Figure 2A and detailed in Supplementary Table 1. Incidence rates from 2020 per state are illustrated in Supplementary Figure 1.

The prevalence of IBD increased significantly from 30.0/100,000 in 2012 to 100.1/100,000 in 2020 (AAPC=14.9%; 95% CI 14.78 - 14.95; p<0.0001). In UC, prevalence rates increased from 15.8/100,000 to 56.5/100,000 (AAPC=16.0%; 95% CI 15.94, 16.17; p<0.0001). In CD, prevalence rates increased from 12.6/100,000 in 2012 to 33.7/100,000 in 2020 (AAPC=12.1%;95% CI 11.95, 12.22; p<0.0001). In IBDU, prevalence rates increased from 1.6/100,000 in 2012 to 9.9/100,000 in 2020 (AAPC=19.1%; 95% CI 18.84, 19.41; p<0.0001). These data are illustrated in Figure 2B and Supplementary Table 2.

**Figure 3:**
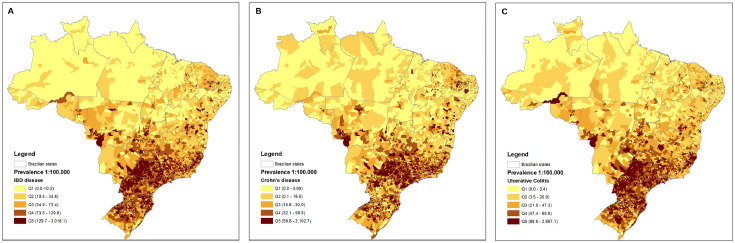
Prevalence rates in 2020 for all 5565 municipalities in Brazil. A: IBD; B: CD and C: UC.

Prevalence data from 5,565 municipalities across all regions of the country were additionally analysed. Figure 3 demonstrates the cities with higher prevalence in IBD, CD and UC. Higher prevalence was observed in the southern and southeastern regions, with cities with lower rates being more commonly observed in the north and northeastern areas. Prevalence rates per each state of the federation are described in Supplementary Figure 2 and Supplementary Table 3. The states of São Paulo (southeastern region), Paraná and Santa Catarina (southern region) had the higher cumulative prevalence rates, whilst the states of Amapá, Pará and Roraima (all from the northern region) presented the lower rates of prevalence from all phenotypes (IBD, CD and UC).

**Figure 4:**
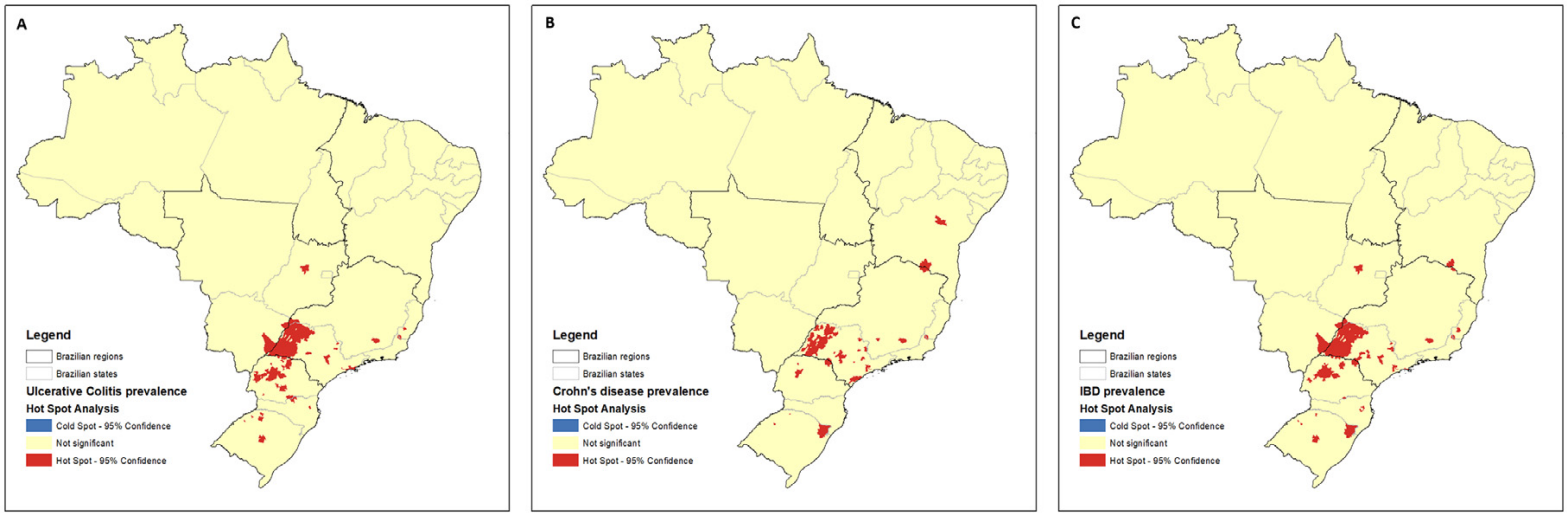
Hot spot analysis of higher prevalence groups of municipalities in Brazil. A: UC; B: CD and C: IBD.

**Figure 5:**
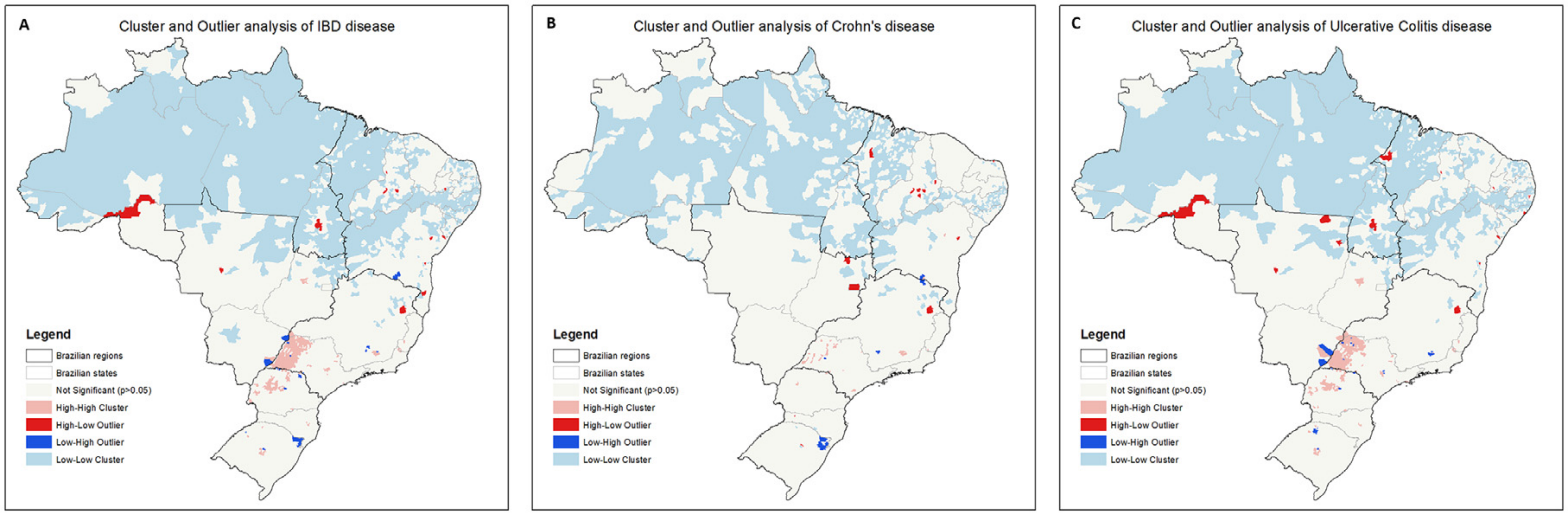
Cluster and outlier analysis of lower prevalence groups of municipalities in Brazil. A: IBD; B: CD and C: UC.

By using the spatial autocorrelation test (Moran's I index), a south-north gradient was significantly described in the 2020 rates of prevalence of IBD [Moran's I=0.40 (p<0.0001)], CD [Moran's I=0.22 (p<0.0001)] and UC [Moran's I=0.42 (p<0.0001)]. In the hot spot analysis, groups of municipalities with higher prevalence (“hot areas”) were more observed in the southern and southeastern regions, mainly in the states of Paraná, west of São Paulo and Rio Grande do Sul, in all phenotypes (Figure 4). In the cluster and outlier analysis, groups of municipalities with lower prevalence rates (“cold areas”) were clearly more distributed in the northern and northeastern areas, mostly in the states of Amazonas, Pará and Amapá (Figure 5).

## Discussion

In this nationwide population-based study from Brazil incidence rates of IBD remained stable over a decade, whilst it slightly decreased in CD and increased in UC. Prevalence significantly increased for IBD, CD and UC. A south-north gradient of prevalence of IBD was observed in 2020, with clusters of cities with higher prevalence being more concentrated in the south and southeastern regions (more developed urbanized areas), whereas groups of cities with lower prevalence rates were more concentrated in the northern and northeastern regions (more rural areas). In 2020, 0.1% of Brazilians are living with IBD contributing to a significant burden to the public healthcare system of Brazil.

There was a predominance of females (58.5%) compatible with what is observed in the international literature.^22^^,^^23^ The 0.5% of patients with undetermined sex were possibly patients with both sexes in different consultations, as fulfilled in forms by treating physicians, patients with change of sex during the course of IBD or transgenders. The majority of patients had a diagnosis of UC, with a UC:CD ratio of 1.7:1. This ratio was slightly higher than what was previously described in a systematic review of epidemiological publications from Latin American countries (overall Brazilian UC:CD ratio of 1.081, varying from 0.481 – 1.936).^7^ This difference is likely related to the fact that previous populations in that systematic review were from medical and surgical referral centres thereby invoking referral bias. On the other hand, the current Brazilian UC:CD ratio was lower than what was observed in other Latin American countries such as Argentina (4.308), Colombia (5.837) or Mexico (4.798).^7^ A recent cohort study from Cordoba, Argentina, reported a UC:CD ratio of 6.38.^24^ Even having more UC cases than CD, the ratio in Brazil is lower than other countries from Latin America, what deserves special attention in future research, and possible reasons for the higher number of CD cases in Brazil as compared to other countries from the same continent can be speculated.

Our results demonstrated that the incidence of CD decreased by 3.2% per year from 3.7/100,000 in 2012 to 2.7/100,000 in 2020. Numbers of 2020 could be probably affected by the pandemic, when most healthcare resources were directly allocated to tackle the COVID-19 outbreak and its consequences, which could account for this reduction.^25^ Mental stress and different diet regimens during the pandemic could theoretically be related to the onset of new IBD diagnoses. The decrease in CD incidence rates in 2020 could be related purely to patients not having access to public healthcare. Future national studies emphasizing numbers in the pandemic period are warranted to explain the real impact of COVID-19 in IBD incidence and prevalence rates in Brazil.

On the other hand, incidence of UC significantly increased by 3% per year from 5.7/100,000 to 6.9/100,000 in the same period. This tendency is consistent with observations from other countries in Latin America. A national registry from Colombia demonstrated that UC incidence increased significantly from 5.59/100,000 in 2010 to 6.3/100,000 in 2017, numbers which are similar to our current findings in Brazil.^26^ A Mexican study demonstrated similar trends, with an increase in crude incidence rates in UC from 2005 to 2015.^27^

These up-to-date epidemiological data highlight the rising burden of IBD in Brazil. The nationwide prevalence of IBD in Brazil steadily climbed to 0.1% of the population in 2020. Countries in the Western world are currently in the third epidemiological stage of ‘Compounding Prevalence’ whereby incidence stabilizes, but prevalence continues to climb.^3^^,^^28^ Data from Canada and Scotland have reported the prevalence of IBD to be approximately 0.7% in 2020.^29^^,^^30^ The stabilization of incidence in Brazil alongside the rapidly climbing prevalence is consistent with western countries and highlights that Brazil may be transitioning to the Compounding Prevalence Epidemiological Stage.

Our prevalence in 2020 (IBD: 100.1/100,000 UC: 56.5/100,000; CD: 33.7/100,000; and IBDU: 9.9/100,000) have updated what was previously considered in global reviews.^2^ These numbers put Brazil as a country with intermediate to high prevalence of IBD in the 21st century. IBD management and access to healthcare in our country improved significantly over the last decades.^31^^,^^32^ Prompt access to biological therapy, early treatment and multidisciplinary management are common practices in different regions of the country. Patients are usually young when they enter the public system with an IBD diagnosis. They also have access to better care, and as a consequence, mortality is reduced, what contributes to the accumulation of cases throughout the country.

A previous study with data derived from the same database demonstrated similar findings in terms of incidence and prevalence of IBD in Brazil, with inclusion of 162,894 patients from 2008 to 2017.^14^ Our study included more patients (n=212,026) in a more recent period (2012–2020). One of the main differences between our recent analysis and this previous study is based on our use of temporal analysis with AAPC, specific statistical analyses to demonstrate potential gradients between different regions and consideration of UIBD in patients with both diagnosis (UC and CD), what may solidify our findings.

One of the most interesting findings of our national study is the significant gradient in prevalence rates between the different regions of the country in 2020. Clusters of cities with higher prevalence of IBD were more concentrated in southern and southeastern regions, whilst cities with lower prevalence rates were more distributed in the northern and northeastern regions, with the Moran's index, hot spot and cluster and outlier analyses. This gradient confirms what was demonstrated in previously published studies from Brazil. In the state of São Paulo and Espírito Santo, prevalence rates of IBD were 52.6/100,000 and 38.2/100,000, respectively.^11^^,^^12^ A study from the state of Piauí, in the northern part of the country, with lower HDI, demonstrated prevalence rates of IBD of only 12.8/100,000.^10^ Our study confirms these differences, which were studied only locally in previous analyses.

Several factors could justify these findings. First, states from the south and southeast comprise more developed areas, with higher indexes of urbanization and higher HDI.^15^ Secondly, these regions were colonized by different immigrants in the 19th century, mostly Europeans, what could bring a genetic component for the higher numbers of cases. The northern states of the federation also have more rural areas, and access to medical care can be difficult in these regions. Other factors such as differences in diet or sunlight exposure could also play an additional role on these differences and deserve attention in future research. The same reasons could be speculated when comparing numbers from Brazil with other Latin American countries, as Brazil is the mostly industrialized country in the continent, with a different genetic background.

Our study has some limitations. Numbers were exclusively extracted from the public healthcare system and patients exclusively managed in the private healthcare system were not included and the figures could be an underestimation. However, it is a common practice in Brazil that patients from the private sector eventually move to the public setting due to need for access to expensive medications (e.g., biologics). Hence, the majority of those living with IBD are likely to be captured by our system. ICD codes were also not always included by IBD specialists. Any physician in the country can input these patients into the system. This could bias, for instance, specific ICD codes for the patients, with a switch between diagnosis of UC and CD. Changes in disease phenotype during the study period could also have an impact on our specific numbers. Specific characteristics of CD and UC such as Montreal classification were not captured. Additionally, IBD are not compulsorily notified. Thus, confirmation of the diagnoses by specific healthcare committees was not performed. Access to healthcare is heterogeneous in our country, what could bring lower numbers of IBD diagnoses in areas from the north and northeastern regions. Lastly, other types of colitis or causes of diarrhoea could be labelled as IBD by treating physicians, another possible bias in specific diagnosis.

Despite these limitations, the strengths of our study are based on the large number of included patients and the full national coverage (all states of the federation) of our unique public healthcare system. This is difficult to be found in newly industrialized countries. The use of artificial intelligence to capture these data also overcame important barriers for data analysis in a large population as ours. The specific analyses used for internal differences in prevalence also contributed for bias reduction and improved our results.

In summary, incidence rates of IBD have remained stable in Brazil from 2012-2020. Incidence of CD is significantly decreasing whereas of UC is significantly increasing. The global pandemic may have influenced diagnoses of IBD in 2020 and thus, future epidemiological studies are necessary to investigate an upswing in incidence over the next few years. There was a significant increase in cumulative prevalence rates of CD and UC over the nine years of the study period. Prevalence rates in 2020 were higher in the south and southeastern regions as compared to north and northeastern, determining a south-north gradient of IBD cases in our country.

This significant rise in prevalence can support planning for future strategies for public healthcare providers in our country towards better IBD care. Future research is expected for possible factors associated to the internal differences or increase in prevalence rates, such as diet, genetic background, or other environmental factors. This is the largest IBD epidemiological study from newly industrialized countries to date.

## Contributors

ABQ, GGK and PGK designed the study. ABQ, DAV and PGK collected data. AAFH and SC did statistical analyses and geographical mapping. AOMCD, CSRC, DOM, RP and SCN did data analyses and gave important intellectual contribution for the manuscript. All authors reviewed and approved the final version.

## Data sharing statement

DATASUS is a publicly available database, and can be checked in this link: http://www.datasus.gov.br.

## Editor note

The Lancet Group takes a neutral position with respect to territorial claims in published maps and institutional affiliations.

## Declaration of interests

Quaresma AB has received honoraria for speaking from AbbVie, Apsen and Janssen.

Aderson OMCD has received honoraria for speaking from AbbVie, Janssen, Pfizer, and Takeda; he has been a consultant for Takeda, AbbVie, and Janssen.

Coy CSR has no conflicts of interest.

Magro DO has no conflicts of interest.

Hino AAF has no conflicts of interest.

Valverde DA is CEO of Techtrials a Healthcare Data Science Company.

Panaccione R reports consulting from AbbVie, Abbott, Alimentiv (formerly Robarts), Amgen, Arena Pharmaceuticals, AstraZeneca, Bristol-Myers Squibb, Boehringer Ingelheim Celgene, Celltrion, Cosmos Pharmaceuticals, Eisai, Elan, Eli Lilly, Ferring, Galapagos, Genentech, Gilead Sciences, Glaxo-Smith Kline, Janssen, Merck, Mylan, Oppilan Pharma, Pandion Pharma, Pfizer, Progenity, Protagonist Therapeutics, Roche, Satisfai Health, Sandoz, Schering-Plough, Shire, Sublimity Therapeutics, Theravance Biopharma, UCB, and Takeda Pharmaceuticals. He has received speaker's fees from AbbVie, Arena Pharmaceuticals, Celgene, Eli Lilly, Ferring, Gilead Sciences, Janssen, Merck, Pfizer, Roche, Sandoz, Shire, and Takeda Pharmaceuticals.

Coward SB has no conflicts of interest.

Ng SC has received consulting and speaker fees from AbbVie, Ferring, Janssen, Menarini, and Takeda; served as a Scientific Advisory Board member for AbbVie, Ferring, and Takeda; and received research grants from AbbVie, Ferring, and Janssen.

Kaplan GG has received honoraria for speaking or consultancy from AbbVie, Janssen, Pfizer, Amgen, and Takeda. He has received research support from Ferring, Janssen, AbbVie, GlaxoSmith Kline, Merck, and Shire. He has been a consultant for Gilead. He shares ownership of a patent: TREATMENT OF INFLAMMATORY DISORDERS, AUTOIMMUNE DISEASE, AND PBC. UTI Limited Partnership, assignee. Patent WO2019046959A1. PCT/CA2018/051098. 7 Sept. 2018.

Kotze PG has received consulting and speaker fees from AbbVie, Janssen, Pfizer, and Takeda. He also received scientific grants from Pfizer and Takeda.

## Appendix Supplementary materials

Image, application 1

Image, application 2

Image, application 3

Image, application 4

Image, application 5
