# Supplementary material for: Temporal trends in the epidemiology of inflammatory bowel diseases in the public healthcare system in Brazil: A large population-based study
Source: Lancet Reg Health Am. 2022 Jun 9;13:100298. doi: 10.1016/j.lana.2022.100298 (PMC9903988; doi:10.1016/j.lana.2022.100298)
Supplement: Supplementary file 1 [file mmc1.docx]

**SUPPLEMENTARY MATERIAL:**

| **Year** | **New IBD cases** | **IBD incidence** | **New CD cases** | **CD incidence** | **New UC cases** | **UC Incidence** | **Estimated population*** |
| --- | --- | --- | --- | --- | --- | --- | --- |
| 2012 | 18,244 | 9.41 | 7,203 | 3.71 | 11,041 | 5.69 | 193,946,886 |
| 2013 | 17,326 | 8.62 | 6,871 | 3.42 | 10,455 | 5.20 | 201,032,714 |
| 2014 | 18,899 | 9.32 | 7,642 | 3.77 | 11,257 | 5.55 | 202,768,562 |
| 2015 | 18,353 | 8.98 | 7,155 | 3.50 | 11,198 | 5.48 | 204,450,649 |
| 2016 | 18,204 | 8.83 | 6,635 | 3.22 | 11,569 | 5.61 | 206,081,432 |
| 2017 | 19,016 | 9.16 | 6,597 | 3.18 | 12,419 | 5.98 | 207,660,929 |
| 2018 | 18,178 | 8.72 | 6,413 | 3.08 | 11,765 | 5.64 | 208,494,900 |
| 2019 | 21,538 | 10.25 | 6,776 | 3.22 | 14,762 | 7.02 | 210,147,125 |
| 2020 | 20,257 | 9.57 | 5,666 | 2.68 | 14,591 | 6.89 | 211,755,692 |

**Supplementary table 1: Data on the incidence of IBD in Brazil from 2012 to 2020.**

**Incidence is number / 100,000 inhabitants. *Source: IBGE.**
