# Supplementary material for: Temporal trends in the epidemiology of inflammatory bowel diseases in the public healthcare system in Brazil: A large population-based study
Source: Lancet Reg Health Am. 2022 Jun 9;13:100298. doi: 10.1016/j.lana.2022.100298 (PMC9903988; doi:10.1016/j.lana.2022.100298)
Supplement: Supplementary file 2 [file mmc2.docx]

| **Year** | **IBD cases** | **IBD prevalence** | **CD cases** | **CD prevalence** | **UC cases** | **UC prevalence** | **IBDU cases** | **IBDU prevalence** | **Estimated population*** |
| --- | --- | --- | --- | --- | --- | --- | --- | --- | --- |
| 2012 | 58,202 | 30.01 | 24,447 | 12.61 | 30,588 | 15.77 | 3,167 | 1.63 | 193,946,886 |
| 2013 | 76,570 | 38.09 | 30,736 | 15.29 | 40,055 | 19.93 | 5,779 | 2.87 | 201,032,714 |
| 2014 | 96,051 | 47.37 | 37,602 | 18.54 | 50,051 | 24.68 | 8,398 | 4.15 | 202,768,562 |
| 2015 | 114,717 | 56.11 | 43,342 | 21.20 | 60,109 | 29.40 | 11,266 | 5.51 | 204,450,649 |
| 2016 | 133,120 | 64.60 | 49,028 | 23.79 | 70,714 | 34.32 | 13,378 | 6.49 | 206,081,432 |
| 2017 | 152,280 | 73.33 | 54,627 | 26.03 | 81,982 | 39.48 | 15,671 | 7.55 | 207,660,929 |
| 2018 | 170,572 | 81.81 | 60,288 | 28.91 | 92,628 | 44.43 | 17,656 | 8.47 | 208,494,900 |
| 2019 | 192,198 | 91.46 | 66,373 | 31.59 | 106,281 | 50.58 | 19,544 | 9.29 | 210,147,125 |
| 2020 | 212,026 | 100.13 | 71,321 | 33.68 | 119,700 | 56.53 | 21,005 | 9.92 | 211,755,692 |

**Supplementary table 2: IBD prevalence data in Brazil from 2012 to 2020.**

**Prevalence is number/100,000 inhabitants. *Source: IBGE.**
