# Supplementary material for: Temporal trends in the epidemiology of inflammatory bowel diseases in the public healthcare system in Brazil: A large population-based study
Source: Lancet Reg Health Am. 2022 Jun 9;13:100298. doi: 10.1016/j.lana.2022.100298 (PMC9903988; doi:10.1016/j.lana.2022.100298)
Supplement: Supplementary file 3 [file mmc3.docx]

| **State** | **IBD cases** | **IBD prevalence** | **CD cases** | **CD prevalence** | **UC cases** | **UC prevalence** | **IBDU**  **cases** | **IBDU**  **prevalence** | **Estimated population*** |
| --- | --- | --- | --- | --- | --- | --- | --- | --- | --- |
| **PR** | 19,949 | 173.22 | 6,508 | 56.51 | 12,617 | 109.56 | 824 | 7.15 | 11,516,840 |
| **RS** | 12,580 | 110.13 | 3,906 | 34.02 | 7,536 | 65.97 | 1,138 | 9.96 | 11,422,973 |
| **SC** | 10,745 | 148.16 | 3,399 | 46.87 | 6,932 | 95.59 | 414 | 5.70 | 7,252,502 |
| **SP** | 84,623 | 182.81 | 28,047 | 60.59 | 44,843 | 96.87 | 11,733 | 25.35 | 46,289,333 |
| **MG** | 20,350 | 95.75 | 8,347 | 39.38 | 10,383 | 48.94 | 1,620 | 7.43 | 21,292,666 |
| **RJ** | 10,699 | 61.61 | 5,061 | 29.14 | 5,195 | 29.92 | 443 | 2.55 | 17,366,189 |
| **ES** | 4,551 | 111.98 | 1,455 | 35.80 | 2,873 | 70.69 | 223 | 5.49 | 4,064,052 |
| **BA** | 8,063 | 54.00 | 3,222 | 21.58 | 3,367 | 22.55 | 1,474 | 9.87 | 14,930,634 |
| **PE** | 5,818 | 60.50 | 682 | 7.09 | 4,997 | 51.96 | 139 | 1.45 | 9,616,621 |
| **CE** | 4,802 | 52.27 | 2,255 | 24.55 | 1,928 | 20.99 | 619 | 6.73 | 9,187,103 |
| **MA** | 2,055 | 28.88 | 427 | 6.00 | 1,463 | 20.56 | 165 | 2.32 | 7,114,598 |
| **PB** | 2,108 | 52.19 | 671 | 16.61 | 1,194 | 29.56 | 243 | 6.02 | 4,039,277 |
| **RN** | 2,034 | 57.55 | 751 | 21.25 | 1,083 | 30.64 | 200 | 5.66 | 3,534,165 |
| **AL** | 3,040 | 90.70 | 376 | 11.21 | 2,474 | 73.81 | 190 | 5.68 | 3,351,543 |
| **PI** | 1,967 | 59.94 | 729 | 22.21 | 1,118 | 34.07 | 120 | 3.66 | 3,281,480 |
| **SE** | 1,066 | 45.97 | 411 | 17.72 | 583 | 25.14 | 72 | 3.11 | 2,318,822 |
| **PA** | 1,059 | 12.19 | 263 | 3.03 | 740 | 8.52 | 56 | 0.64 | 8,690,745 |
| **AM** | 1,281 | 30.44 | 201 | 4.77 | 1,002 | 23.81 | 78 | 1.86 | 4,207,714 |
| **RO** | 928 | 51.66 | 158 | 8.80 | 729 | 40.58 | 41 | 2.28 | 1,796,460 |
| **TO** | 509 | 32.01 | 203 | 12.77 | 286 | 17.99 | 20 | 1.25 | 1,590,248 |
| **AC** | 267 | 29.85 | 28 | 3.13 | 226 | 25.27 | 13 | 1.45 | 894,470 |
| **AP** | 48 | 5.57 | 16 | 1.86 | 29 | 3.37 | 3 | 0.34 | 861,773 |
| **RR** | 138 | 21.86 | 71 | 11.24 | 53 | 8.39 | 14 | 2.23 | 631,181 |
| **GO** | 6,854 | 96.35 | 2,071 | 29.11 | 4,280 | 60.17 | 503 | 7.07 | 7,113,540 |
| **MT** | 2,342 | 66.42 | 778 | 22.07 | 1,340 | 38.00 | 224 | 6.35 | 3,526,220 |
| **DF** | 3,450 | 112.92 | 1,318 | 43.14 | 2,022 | 66.18 | 110 | 3.60 | 3,055,149 |
| **MS** | 2,597 | 92.44 | 992 | 35.31 | 1,382 | 49.19 | 223 | 7.94 | 2,809,394 |

**Supplementary table 3: prevalence rates of IBD, CD, UC and IBDU by all different Brazilian states in 2020.**

**Subtitles: PR: Paraná, RS: Rio Grande do Sul, SC: Santa Catarina, SP: São Paulo, MG: Minas Gerais, ES: Espírito Santo, Ba: Bahia, PE: Pernambuco, CE: Ceará, MA: Maranhão, PB Paraíba, RN: Rio Grande do Norte, AL: Alagoas, PI: Piauí, SE: Sergipe, PA: Pará, AM: Amazonas, RO: Rondônia, TO: Tocantins, AC: Acre, AP: Amapá, RR Roraima, GO: Goiás, MT Mato Grosso, DF: Distrito federal, MS: Mato Grosso do Sul.**

**Prevalence is number/100,000 inhabitants.**

*** Source: IBGE.**
