# Supplementary material for: Temporal trends in the epidemiology of inflammatory bowel diseases in the public healthcare system in Brazil: A large population-based study
Source: Lancet Reg Health Am. 2022 Jun 9;13:100298. doi: 10.1016/j.lana.2022.100298 (PMC9903988; doi:10.1016/j.lana.2022.100298)

**Supplementary figure 1: incidence rates in 2020 by state of the federation. A: IBD; B: CD and C: UC. Incidence is number/100,000 inhabitants.**


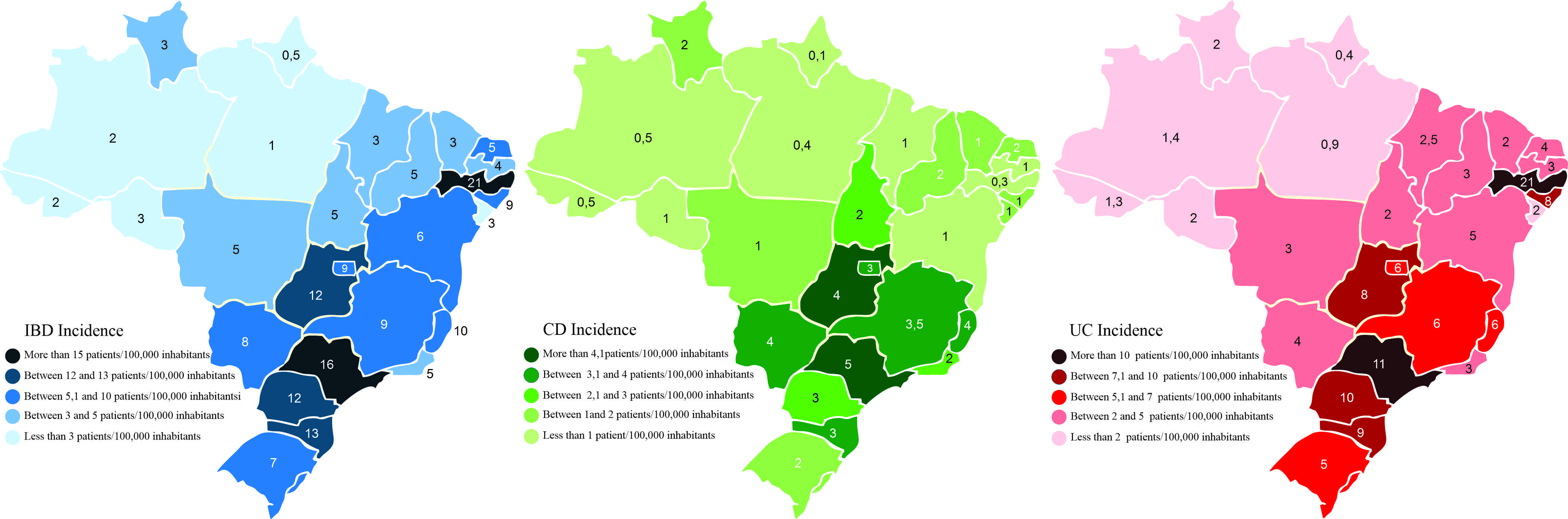

Supplement: Supplementary file 4 [file mmc4.docx]
